# Three years mortality analysis in general surgery patients

**DOI:** 10.12669/pjms.37.1.2040

**Published:** 2021

**Authors:** Dileep Kumar, Hina Bukhari, Shamim Qureshi

**Affiliations:** 1 Dr. Dileep Kumar, Associate Professor, Department of General Surgery, Ward-2, Jinnah Postgraduate Medical Center, Karachi, Pakistan; 2 Dr. Hina Bukhari, Senior Registrar, Department of General Surgery, Ward-2, Jinnah Postgraduate Medical Center, Karachi, Pakistan; 3 Prof. Dr. Shamim Qureshi, Department of General Surgery, Ward-2, Jinnah Postgraduate Medical Center, Karachi, Pakistan

**Keywords:** Surgical, Mortality, Patients, Complications, Sepsis, Pulmonary embolism, Myocardial infarction

## Abstract

**Objective:**

Surgical patient mortality is progressively being measured for providing better management and care in different healthcare systems world-wide. The aim of this study was to assess mortality within a surgical department and to evaluate components associated with surgical and non- surgical deaths.

**Methods:**

We retrospectively collected data including all admissions, both operative and non-operative, surgical procedures and reason of admission (for non-operative) and mortalities within three-year period (2015-2017) from Surgical Unit-2, JPMC Karachi. We assessed type of operations, admission, patient related factors including age, sex, co-morbid factors, reason, time and onset of presentation, operative notes, events, clinical cause and date/ time of death.

**Results:**

The total admissions of 5730 patients were observed in surgical ward-2 during the period of 1^st^ of January 2015 and 31^st^ of December 2017. There were a total of 291 deaths during this period (5.07% overall mortality rate). GIT related (peritonitis/ obstruction) (58.41%), biliarytract/ pancreatic causes (10.9%), road traffic accidents/ blunt trauma (7.21%), firearm injuries (1.71%) followed by GIT malignancies (4.81%) and Non-GIT malignancies (2.06%) were observed to be the main/ leading causes of death. Of the 291 deaths, males were 179 (6.70%) and females were 112 (3.66%). Male to female ratio of morality came out to be 1.6:1. The cause of death in our patients was sepsis (58.41%), cardiopulmonary arrest (13.0%), trauma/ gunshot injuries 8.93%, advanced malignancies (6.87%), pulmonary embolism (6.18%), myocardial infarction (5.49%) and post op bleeding (1.03%). Mortality due to delayed presentation of patient i.e. after five days of onset of symptoms (62.88%), Surgical decision/ exploration after 24 hours (33.67%). The lack of availability of ICU/ HDU in hospital contributed (51.01%) to the total surgical mortality.

**Conclusions:**

As per the study of three years (2015-2017) a fluctuating mortality pattern is observed. The increment of death was mainly among the unavoidable deaths such as GIT and Non GIT related sepsis, advanced malignancies, trauma and firearm injuries, pulmonary embolism myocardial infarction, a moderate role has also been played by miscellaneous group of patients. Delayed presentation of the patients after appearance of first symptom/ symptoms, delayed surgical decision/ exploration also came out to be significantly important factors in our studies elaborating the major difference in mortality rate.

## INTRODUCTION

Reduction in death rate is a primary responsibility of treating doctor and timely decision of the surgeon can reduce the death rate of the surgical patients. The audit of mortalities in surgical patients provide an opportunity to the surgeons, to identify the basic causes of death, in order to modify the ongoing policies and to forestall the events leading to avoidable deaths in surgical wards.^1^-^3^ It helps in identifying various reasons related to pathology, burden of the disease, role of co-morbidities and complications of the operative procedure. It further helps in modifying or changing the surgical strategies in addition to provide more care where needed identifying the loop holes in surgical practice and to fill the gaps in a specific surgical care.

While European and western countries have done multiple studies in this regard, we noticed that the work done regarding surgical mortality analysis in developing countries is negligible. Like, work done by Nixon SJ, Krishnamurthy VR et al, and Vallabha T et al have shown mortality rate in the surgical wards as 9.14%, 5.09%, 16.1% and 4.2% respectively.^2^-^4^

The objective of this study was to assess the mortalities in our surgical ward and role of audit in changing surgical practice. The results obtained from this study will help to correct the causes behind avoidable deaths in terms of educating and creating awareness among the surgical professionals to improve the already set standards of quality of care about preventable deaths and to change, modify and strengthen the surgical setup and policies.^4^,^5^

## METHODS

A retrospective descriptive observational study undertaken on all the patients admitted to the Surgical Ward-2 Jinnah Postgraduate Medical Center (JPMC) in Karachi, Sindh Pakistan. The study period as from 1^st^ January 2015 to 31^st^ December 2017. The hospital is a tertiary care 1600 bedded government teaching hospital. Retrospectively all the patients admitted to surgery Ward-2 in study period of three years were included in the audit/ study. We took the approval from the ethical committee on 18 November, 2017. (Ref.F.56/admin/jpmc).

Data for this study was collected from the medical record room of JPMC. Records were collected from ward registers and case notes of all elective or emergency admissions during the study period. A three hours of hospitalization at minimum was required to be an inclusive patient. A Performa sheet was used to organize the collected data which included age, sex, diagnosis of patient, co-morbid factors, time of presentation after initiation of symptoms, surgery performed, events leading to death and clinical cause of death. The patients who were brought dead or died within emergency room immediately were excluded. Delayed Presentation was taken as more than five days from appearance of first symptom for emergency patients (advanced malignancies do not fall in this group of patients). Delayed decision/ exploration is taken as decision/ exploration taken more than 24 hours after presentation to the hospital. Only two patients were re-admitted out of total patients taken into consideration. Furthermore, no autopsy was done/ included in this study. The traditional case definition of in-hospital surgical mortality of deaths occurring within 30 days of admission for surgical care was included in study.^6^

## RESULT

During the period of 2015-2017 there were 5730 admissions to our surgical ward including 2670 males and 3060 females. There were a total of 291 deaths during this period (5.07% overall mortality rate) with female mortality as (3.66%) and male mortality rate as (6.70%) (Table I and II). GIT related (peritonitis/ obstruction) (58.41%), biliary tract/ pancreatic causes (10.9%), road traffic accidents/ blunt trauma (7.21%) firearm injuries (1.71%), followed by GIT malignancies (4.81%) and Non-GIT malignancies (2.06%) were observed to be the main/ leading causes of death. Of the 291 deaths, males were 179 (6.70%) and females were 112 (3.66%). Male to female ratio of morality came out to be 1.6:1 (Table-III).

**Table-I:** Gender specific mortality rate.

Year	Admissions	Male admissions	Male mortality	Mortality rate in males %	Female admissions	Female mortality	Mortality rates in females %	Total mortalities n	Total mortality rate %

2015	1973	887	61	6.8%	1086	36	3.31%	97	4.91%
2016	1744	800	38	4.75%	944	28	2.96%	66	3.78%
2017	2013	983	80	8.13%	1030	48	4.66%	128	6.35%

Total	5730	2670	179	6.70%	3060	112	3.66%	291	5.07%

**Table-II:** Age wise mortality rate during the study period (2015-2017).

Age in years	Male deaths	Female deaths	Total deaths	Percentage%

13-20	10	7	17	5.84%
20-30	13	10	23	7.90%
30-40	35	24	59	20.2%
40-50	41	25	66	22.68%
50-60	21	34	55	18.90%
60-70	36	12	48	16.49%
70-80	15	8	23	7.90%

Total	171	120	291	100

**Table-III:** Pathological reasons specific to death rate.

Diagnosis of patient	2015	2016	2017	Total patients

GIT related (peritonitis/obstruction)	53	44	73	170
Biliary tract/ pancreatic cause	12	6	14	32
Blunt trauma	9	5	7	21
Firearm	2	1	2	5
G.I malignancies	4	3	7	14
Other malignancies	3	1	2	6
Miscellaneous	14	6	23	43

Total	97	66	128	291

The common reason for death were severe systemic sepsis (58.41%), cardiopulmonary arrest (13.0%), trauma/ gunshot injuries 8.93%, advanced malignancies (6.87%), pulmonary embolism (6.18%), myocardial infarction (5.49%) and post-op. bleeding (1.03%) (Table-IV).

**Table-IV:** Disease related causes of mortality (2015-2017).

Disease related causes		

Sepsis	170	58.41%
Cardiopulmonary arrest	38	13.0%
Trauma/ gunshot	26	8.93%
Advanced malignancy	20	6.87%
Pulmonary embolism	18	6.18%
Myocardial infarction	16	5.49%
Bleeding	3	1.03%

Mortality due to delayed presentation of patient (62.88%), delayed surgical decision/ exploration after 24 hours (33.67%). The lack of availability of ICU/ HDU in hospital contributed (51.01%) to the total surgical mortality (Table-V).

**Table-V:** Other causes of surgical mortality.

Early presentation of patient	108	37.11%
Delayed presentation	183	62.88%
Decision/ exploration within 24 hours	193	66.32%
Decision/ exploration after 24 hours	98	33.67%

## DISCUSSION

Surgical patient deaths are a very important point of concern for surgeons, hospital death leaves a grievous impact on the patient’s family and surgeons themselves. It highly affects surgeon’s occupational moral especially when the death occurs out of an avoidable cause.^6^

The whole purpose of carrying out this study was to learn from our flaws in surgical management, improve the surgical care system, to introduce and imply new policies in health care systems in order to reduce the avoidable mortality rate. According to Godale et al and Kulkarni SK et al the continuous review/ audits of the surgical and anesthesia practice leading to surgical mortality will help alleviating the adverse outcome in surgical practice and strengthening the surgical practice and reduce the burden of mortality.^6^,^7^ In the time period from 2015-2017 the total surgical mortality rate of 5.07% is observed in our study. These results co-relate to some extent to studies from authors Hayat WW et al, Ihegihu CC et al, 6.2%, 6.4% respectively.^5^,^8^

The absolute number of deaths show mixed pattern of crust and troughs along with the varying trends in the crude death rates with the similar fluctuating trend in the mortality rate as well. The same very trend has been observed among the surgical patients regarding mortality rate in many of the studies.^8^,^9^ Male to female mortality rate is 6.70% to 3.66% respectively showing male deaths to be double the female deaths. Gender specificity of the mortality rate is observed in a similar pattern in many studies as in ours.^10^ Two third of the surgical patients admissions are comprise of men as compared to women according to many studies but result of our study show a completely opposite trend.

In our study leading cause of deaths are GIT related sepsis (peritonitis/ obstruction) (58.41%), biliarytract/ pancreatic causes (10.9%), road traffic accidents/ blunt trauma (7.21%), firearm injuries (1.71%) followed by Non septic GIT malignancies (4.81%) and Non-GIT malignancies (2.06%). Of the 291 deaths males were 179 (6.70%) and females were 112 (3.66%).^10^ Sepsis contributed (58.41%), cardiopulmonary arrest (13.0%), trauma/ gunshot injuries 8.93%, advanced malignancies (6.87%), pulmonary embolism (6.18%), myocardial infarction (5.49%) and post op. bleeding (1.03%).^11^,^12^

While taking the age specific mortality into consideration third, fourth and fifth decades showed the maximum rate of mortality as [20.9%-22.68%] with the peak mortality in fourth decade of life while male deaths peaking 5^th^ decade of life. This trend is also observed in many studies as well. Hence, the peak of surgical deaths occurs at least a decade earlier then the deaths in medical patients. The malignancy and co-morbid factors contribute to these Cond peak of death in fifth decade. The miscellaneous category includes family neglected patients (14.17%), which also contribute to the increasing pattern of mortality. Mortality due to delayed presentation of patient i.e. after 5 days of onset of symptoms (62.88%).^12^ Surgical Decision/exploration after 24 hours (33.67%).^13^ The lack of availability of ICU/ HDU in hospital contributed (51.01%) to the total surgical mortality. These results of our study are comparable with some other international studies.^13^,^14^

As to sum up regular mortality audits and accurate documentation is in high need of improvement, computerized data saving should be considered in healthcare systems including age, sex, residence of patient, co-morbid factors, reason, time and onset of presentation, operative notes, events, clinical cause and date/ time of death. The national health policies regarding the surgical care and general health care system including the adequate availability of ICU/ HDU, timely availability of investigation for emergency patients, adequate availability of operating theaters, should be reviewed by the responsible administrative/ government authorities with a representative of a surgical team. Furthermore, the patient literacy level, poverty and provision of healthcare play directly proportional to each other for which the policies should be made by the government authorities in collaboration with healthcare providers.^15^-^17^

### Limitations of the study

The documentation errors, retrospective form of study, missing number of files, incomplete details may be considered as limitation of the study, which was tried to be overcome as much as possible. The authorities of different hospitals are usually referring the patients late were contacted and requested through proper channel to refer patients in time to save lives. Patients education regarding the disease process and general healthcare was done as per the hospital policy but needs to be worked on, for which whole new studies should be conducted.

### Authors’ Contribution:

**DK** conceived, designed, did data collection, & editing of manuscript.

**HB** did manuscript writing and statistical analysis.

**MSQ** did review and final approval of manuscript.

**DK** takes the responsibility and is accountable for all aspects of the work in ensuring that questions related to the accuracy or integrity of any part of the work are appropriately investigated and resolved.
